# Solution-processed germanium nanowire-positioned Schottky solar cells

**DOI:** 10.1186/1556-276X-6-287

**Published:** 2011-04-04

**Authors:** Ju-Hyung Yun, Yun Chang Park, Joondong Kim, Hak-Joo Lee, Wayne A Anderson, Jeunghee Park

**Affiliations:** 1 Nano-Mechanical Systems Research Center, Korea Institute of Machinery and Materials (KIMM), Daejeon 305343, Korea; 2 Department of Electrical Engineering, University at Buffalo, State University of New York, Buffalo, New York 14260, USA; 3 Measurement and Analysis Division, National Nanofab Center (NNFC), Daejeon 305806, Korea; 4 Department of Chemistry, Korea University, Jochiwon 339700, Korea

## Abstract

Germanium nanowire (GeNW)-positioned Schottky solar cell was fabricated by a solution process. A GeNW-containing solution was spread out onto asymmetric metal electrodes to produce a rectifying current flow. Under one-sun illumination, the GeNW-positioned Schottky solar cell yields an open-circuit voltage of 177 mV and a short-circuit current of 19.2 nA. Schottky and ohmic contacts between a single GeNW and different metal electrodes were systematically investigated. This solution process may provide a route to the cost-effective nanostructure solar architecture.

## Introduction

Nanostructures, such as carbon nanotubes and nanowires, have emerged as potential building blocks in nanoscale applications of microscopy tips [1], gas sensors [2], nanoscale interconnects [3], and field emitters [4] mainly because of their tiny size satisfying the needs of down-scale schemes, efficiently and effectively.

Recently, semiconducting nanowires have been intensively investigated for the production of cost-effective solar cells [5]. This type of nanostructure provides advantages of a short length of carrier collection [5,6] and enhancement of the optical absorption in comparison with the bulk structure [7]. Although the advantages of nanowire-utilizing solar cells have been predicted, their promising potential has not been much achieved because of the difficulty of the junction formation in the tiny nanostructure. The Schottky contact of the metal-semiconductor has attracted huge interests for applications, such as the UV detectors, nanogenerators, and solar cells [8-10] because of the ease of junction formation of the semiconducting nanostructures.

Germanium (Ge) is compatible with Si technology and thus may enhance the performance of Si-based solar cells by modulation of the bandgap to optimize the solar spectrum harvest. Although various nanomaterials have been investigated for solar cell applications [11-13], few researches have been performed on Ge nanowires (GeNWs) for solar cells to date. From the perspective of production, the prescribed solution process is required to achieve cost-effective nanostructure solar cells [10].

We present here the solution-processed GeNW-positioned Schottky solar cell. A GeNW-containing solution was dropped onto a metal electrode. Asymmetric metals were applied to form a Schottky contact, establishing a rectifying current flow. Under light illumination, the contact system of the GeNW to asymmetric metal electrodes provides Schottky solar cell performance. The junction of metal and GeNW using symmetric and asymmetric metal contacts is also systematically investigated.

## Experimental

The growth of GeNWs was achieved by the thermal vapor transport. A 5-nm-thick Au film coated-Si substrate was placed close to a basket containing Ge powder (Germanium 99.98%, Korea Sigma-Aldrich) inside a quartz tube reactor. The growth temperature was controlled at 800°C under an Ar atmosphere.

To prepare NW-containing solution, the GeNW-grown sample was placed in a methanol-filled vial. An ultrasonication process was carried out for 60 min to separate the grown GeNWs from the substrate and then centrifuged at 10000 rpm for 60 min to remove residuals. The GeNW-containing solution of 2 μL was dropped onto metal electrodes under an ac electric field to align the GeNWs between the electrodes. A field-emission scanning electron microscope (FEI Sirion) was used for observing the GeNWs and their positioning on the metal electrodes. A field-emission transmission electron microscope (TEM) (FEI Tecnai F30 Super-Twin) analysis was performed to verify the nanowire structure. Selected-area electron diffraction and fast Fourier transformation (FFTs, GATAN) revealed a crystalline structure and the growth direction of the GeNW.

## Results and discussion

**Figure 1:**
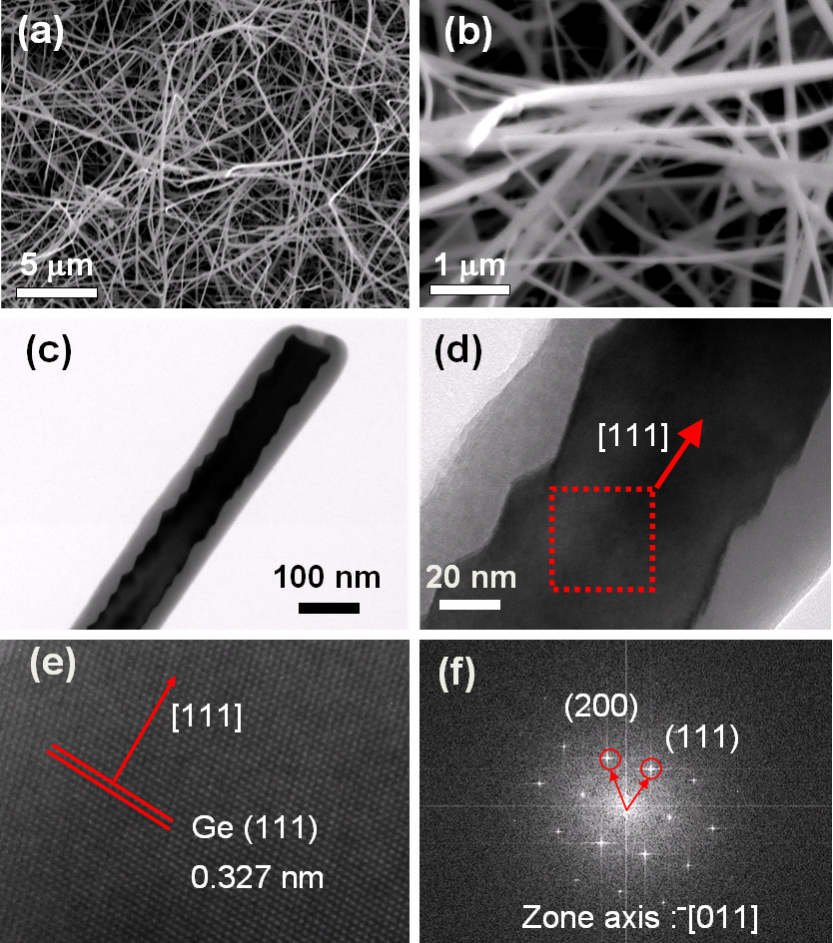
**Observations of the GeNW**. **(a) **Low-magnification SEM image of as grown GeNWs. **(b) **High-magnification SEM image of GeNWs 40-80 nm in diameter. **(c) **TEM image of a single GeNW. **(d) **The HRTEM image shows that the GeNW was grown in the [111] direction. **(e) **The lattice was spaced at 0.327 nm corresponding to Ge (111). The inset is a diffractogram obtained from the HRTEM image.

Typical morphologies of GeNWs grown by the thermal vapor transport method are shown in Figure 1a,b. The length of the nanowires is generally above 10 μm with 40-80 nm in diameter. A TEM image of a single GeNW is shown in Figure 1c. A thin Ge oxide coating was observed, which may be formed during the growth of nanowires. High-resolution TEM (HRTEM) images are presented in Figure 1d,e. The lattices were spaced at 0.327 nm, corresponding to the Ge (111). Figure 1f shows a diffractogram obtained from an HRTEM image with the electron beam parallel to the [011] zone axis, indicating single crystallinity of the GeNW.

**Figure 2:**
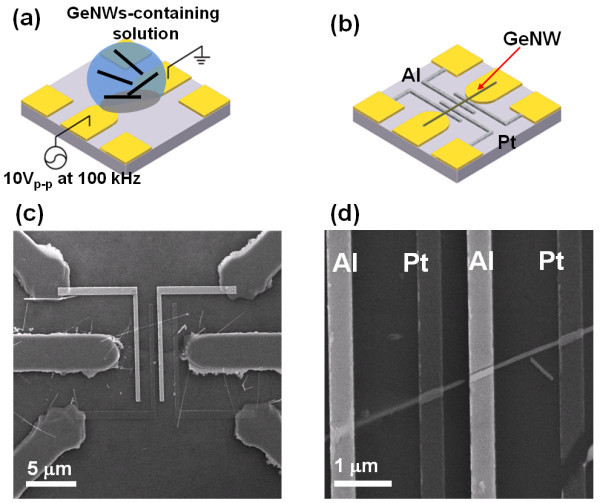
**Schottky junction diode for single GeNW**. **(a) **A schematic diagram of the three-pairs of Ti metal platforms. The solution containing GeNWs was dropped while the centrally located metal pairs were ac-biased to position the GeNWs in the designated location. **(b) **The configuration of the e-beam processed Al and Pt electrodes on the GeNW. **(c) **SEM image of the configuration. **(d) **An enlarged image of **(c) **showing the GeNW alternating contact with the Pt and Al electrodes.

To obtain the electrical connection of a single GeNW, three-pairs of titanium (Ti) metal platforms were prepared by an optical lithography process, as depicted in Figure 2a. The single-pair of Ti electrodes was biased with an ac signal of 10 V_P-P _at 100 kHz, and then 2 μL of the GeNW-containing solution was dropped onto the electrode, thereby positioning the GeNWs in the designated location. Two different metal fingers of Pt and Al were then e-beam patterned and connected to Ti metal platforms. The configuration is presented in Figure 2b, and scanning electron microscope (SEM) images are shown in Figure 2c,d. A single GeNW was alternatively connected to Al and Pt finger electrodes. This feature provides three-different metal contacts to the GeNW. Homogeneous metal connections (Al-GeNW-Al or Pt-GeNW-Pt) and an asymmetric metal connection (Al-GeNW-Pt) were simultaneously formed.

**Figure 3:**
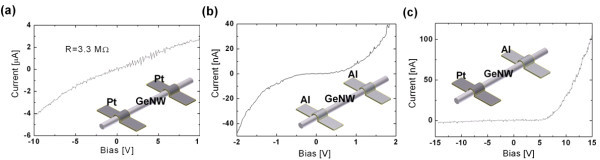
**Current-voltage characteristics in dark condition**. The dark *I*-*V *characteristics of **(a) **Pt-GeNW-Pt, **(b) **Al-GeNW-Al, and **(c) **Pt-GeNW-Al. Ohmic current flows were achieved from the symmetric metal contacts while the asymmetric Pt-Al contacts provide a rectifying current flow due to the Schottky junction.

The dark *I*-*V *characteristics of a single GeNW were obtained from the three different systems. The intrinsic Ge has a work function of 4.33 eV, which is higher than that of Al (4.06 eV) and lower than that of Pt (5.6 eV). The ohmic current flows were obtained from the symmetric Pt-Pt or Al-Al metal contacts as shown in Figure 3a,b, respectively. Otherwise, the asymmetric Pt-Al metal contacts rectified the current flow, as shown in Figure 3c, which is attributed to the different metal contacts to the GeNW establishing an electron barrier between the Pt metal and GeNW and a hole barrier between the Al metal and GeNW.

A laser light source (10 mW) was utilized to obtain the photoresponse from the single GeNW-positioned Schottky device and provided an open circuit voltage (*V*_oc_) of 0.78 V and a short current (*J*_sc_) of 650 pA, corresponding to 2.24 A/cm^2 ^driven from the light-exposed region. Although this high value is induced from the high laser power source, it nevertheless highlights the potential to generate high current from the GeNW comparing to the previous reports on Si NW [12,13].

In order to evaluate the feasibility of large-scale production of GeNWs as a solar cell, asymmetric metal electrodes were prepatterned by an optical lithography process. This provides a rectifying junction between the metals and the semiconducting NW without an e-beam or a focused ion beam [9,12]. A unit device has 30 interdigitated fingers with a length of 500 μm and a 20-μm width. A finger electrode has a 1-μm gap to the neighboring ones.

**Figure 4:**
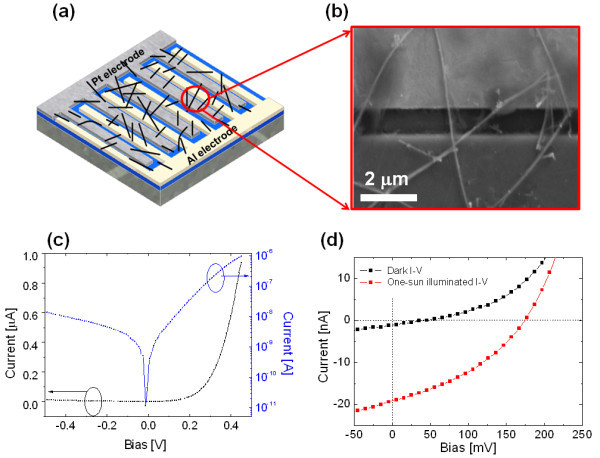
**GeNW-positioned Schottky solar cell**. **(a) **A schematic of the multiple GeNWs Schottky device (Al-GeNWs-Pt). **(b) **SEM image showing the GeNWs in contact with the Pt and Al electrodes. **(c) **The dark *I*-*V *characteristics of the multiple GeNWs device show Schottky diode performance similar to the single GeNW. **(d) **Under one-sun illumination, the Schottky device yields a *V*_oc _of 177 mV and an *I*_sc _of 19.2 nA.

The GeNW-containing solution was spread out onto the interdigitated metal electrodes. A schematic of the multiple GeNW-positioned Schottky device (Al-GeNWs-Pt) is presented in Figure 4a. Figure 4b is a SEM image showing that multiple GeNWs were contacted to the asymmetric metal electrodes.

Under a dark condition, the multiple GeNW-positioned Schottky device clearly provided a diode performance as shown in Figure 4c. Under thermionic emission theory, the *I*-*V *characteristics are given by the following equation:(1)(2)

where *J*_s_, *n*, *kT*, A**, and *ϕ*_B _are the saturation current density, ideality factor, thermal energy (eV), Richardson constant, and barrier height, respectively. The ideality factor was obtained to be 2.37, and the barrier height was calculated to be 0.93 eV.

Under the light condition, the multiple GeNW-positioned Schottky device yields a *V*_oc _of 177 mV and an *I*_sc _of 19.2 nA, as shown in Figure 4d. It is worth noting that the photogenerated voltage is comparable to that of bulk Ge solar cell [14].

The number of GeNWs (*N*) incorporated in the device was calculated by the ratio of the saturation current (*I*_s_) of a single GeNW.(3)

The result indicates that a short-circuit current of 5.8 pA is obtained from a single GeNW, which corresponds to a *J*_sc _of 6.69 mA/cm^2 ^considering the light-exposed area. This value is significantly higher than that (5 mA/cm^2 ^of *J*_sc_) from a single silicon nanowire [13], because the Ge has a smaller bandgap (0.67 eV) with a two-order higher light absorption capability than Si. It demonstrates the potential to enhance the photogenerating current using a GeNW light absorber [14].

## Conclusion

In summary, we have fabricated a solution-processed GeNW-positioned Schottky solar cell. A solution containing GeNWs was spread on metal electrodes under an ac electric field, and the GeNWs were positioned onto the metal electrodes. A Schottky contact between metal and a single GeNW was formed using asymmetric metal electrodes, and the photoresponse was demonstrated under laser light. Under one-sun illumination, the GeNW Schottky solar cell provided an open-circuit voltage of 177 mV and a short-circuit current of 19.2 nA. This solution processed-Schottky solar cell is expected to provide a cost-effective nanostructure solar cell architecture.

## Abbreviations

FFTs: fast Fourier transformation; GeNWs: germanium nanowires; HRTEM: high-resolution TEM; SEM: scanning electron microscope; TEM: transmission electron microscope.

## Competing interests

The authors declare that they have no competing interests.

## Authors' contributions

JHY carried out the fabrication of metal patterns and devices. YCP participated in TEM analysis. JK supervised the experiments and acquired data. HJL performed data analysis. WAA conceived of this study and participated in idea development. JP provided quality GeNWs and important discussion.
